# The G41D mutation in SOD1-related amyotrophic lateral sclerosis exhibits phenotypic heterogeneity among individuals: A case report and literature review

**DOI:** 10.1097/MD.0000000000028771

**Published:** 2022-02-11

**Authors:** Xinyi Zhao, Xinhong Feng, Xiuli Li, Jingyu Mou, Hongjing Liu, Jing Chen, Jian Wu

**Affiliations:** a Department of Neurology, Beijing Tsinghua Changgung Hospital, School of Clinical Medicine, Tsinghua University, Beijing, China; b IDG/McGovern Institute for Brain Research at Tsinghua University, Beijing, China.

**Keywords:** case report, familial amyotrophic lateral sclerosis, genetic sequencing, phenotypic heterogeneity, Cu/Zn superoxide dismutase 1 mutation

## Abstract

**Rationale:**

Amyotrophic lateral sclerosis (ALS) is a progressive and fatal neurodegenerative disease. However, the misdiagnosis of ALS always occurs because of atypical clinical manifestations. Since mutations in Cu/Zn superoxide dismutase 1 (*SOD1*) have been implicated as causative and account for 20% of fALS cases, early genetic sequencing of suspected individuals in ALS pedigrees could be helpful.

**Patient concerns:**

Here we report a Chinese family spanning three generations with fALS. A heterozygous c.125G>A (p.Gly42Asp) missense mutation in exon 2 of *SOD1* gene was detected in our proband as well as her 2 siblings and next generation. Phenotypic diversity was also reported among symptomatic individuals.

**Diagnoses:**

Peripheral blood samples from the proband were collected and sent for polymerase chain reaction (PCR) and Sanger sequencing of the *SOD1* gene at Sanvalley Diagnostics. The other 11 members in the studied family then underwent locus verification.

**Interventions:**

Butylphthalide, Vitamin B12, Coenzyme Q10 and mouse nerve growth factor is given to the symptomatic members.

**Outcomes:**

The symptoms of our proband was not improved by treatments at a late stage. She passed away the fourth year of the disease due to respiratory failure. Two siblings of the proband were given active treatments once verified as carrier. Their symptoms are still limited to limb weakness.

**Lessons:**

This study suggests genetic sequencing is a powerful tool for the diagnosis of familial ALS. Phenotypic heterogeneity exists among G41D-mutated individuals, which further highlights the importance of genomic strategies for early diagnosis.

## 1 Introduction

Amyotrophic lateral sclerosis (ALS) is an adult-onset, fatal neurodegenerative disease characterized by progressive impairment of upper and lower motor neurons.^[1]^ Patients progressively suffer from skeletal muscle weakness, bulbar paralysis, and death due to respiratory failure 3 to 5 years following after onset.^[2]^ Approximately, 5% to 10% of cases are familial ALS (fALS), while sporadic ALS accounts for the remaining cases.^[3]^ Over the past few decades, more than 20 genes have been identified in ALS.^[4]^ Cu/Zn superoxide dismutase (SOD1) is the first reported causative gene^[5]^ that encodes a homodimeric metalloenzyme that catalyzes the conversion of toxic O_2_^-^ into harmless O_2_ and H_2_O_2_.^[6]^ So far, over 185 SOD1 mutations have been implicated and have occurred in 20% of fALS and 2% to 7% of sporadic amyotrophic lateral sclerosis cases.^[7]^ Herein, we report a Chinese family spanning 3 generations with a heterozygous missense mutation (c.125G>A) in exon 2 of the *SOD1* gene. Diverse clinical phenotypes among family members and cases reported in the literature may suggest a complex genotype-phenotype relationship of this mutation. Taking advantage of genetic sequencing, physicians can make a confirmed diagnosis once the disease is onset and provide clinical treatments as early as possible.

## 2 Materials and methods

### 2.1 Pedigree

The pedigree of the studied Chinese family is shown in Figure 1. The proband (II-2), as well as her siblings and offspring, were evaluated according to clinical history, symptoms, or genetic examination. All the participants provided informed consent. The study was approved by the Institutional Ethics Committee of Beijing Tsinghua Changgung Hospital, Beijing, China.

**Figure:**
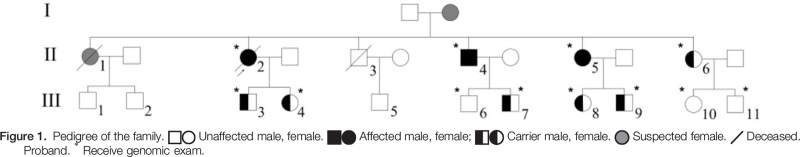


### 2.2 Clinical examinations

The proband (II-2) underwent blood tests, cerebrospinal fluid examination, peripheral nerve ultrasound, electromyography (EMG), brain magnetic resonance scan, and cognitive function evaluation. II-4 and II-5 also underwent EMG.

### 2.3 Genetic approach

Twelve family members (asterisks in Fig. 1) provided peripheral blood samples for molecular genetic testing of SOD1. Polymerase chain reaction (PCR) and Sanger sequencing were performed using Sanvalley Diagnostics.

## 3 Case presentation

Our proband (II-2) was a 58-year-old woman who had progressive muscle weakness in both the upper and lower extremities for 4 years. At the onset, she complained of muscle weakness in her right hand and atrophy of her first interosseous muscle, which was misdiagnosed as cervical spondylosis. Motor and sensory nerve conduction studies suggested peripheral lesions in the 4 limbs. Giant potentials, fibrillation potentials, and positive sharp waves collected from the abductor pollicis brevis; and 1st interosseous muscles by needle EMG indicated neurogenic abnormalities in the bilateral upper extremities. Six months later, she developed muscle weakness in the distal left upper limb. Atrophy of the left 1st interosseous muscle and thenar eminance was also detected. The EMG results suggested carpal tunnel syndrome and neurogenic abnormalities in the 4 limbs.

Without therapeutic intervention, she developed muscle weakness and cramps in both lower extremities (especially the distal right), and the symptoms continued to deteriorate. At the age of 58, she was unable to walk because of severe muscle weakness and atrophy. Neurological examination suggested glossal fasciculation but no dysphagia, dysphonia, or dysarthria. Muscle weakness was noted in both of the upper and lower limbs: Grade V-/V in the left and proximal upper right extremities, Grade III/V in the distal upper right, and Grade I/V in the lower right extremity. Diffused muscle atrophy was detected in the bilateral thenar eminance, 1st interosseous muscles, quadriceps femoris, and tibialis anterior. The biceps and triceps tendon reflexes were hyperactivated bilaterally. Pathological reflections of Hoffman's sign and Rossolimo's sign were elicited. EMG studies suggested peripheral lesions and neurogenic damage in both the upper and lower extremities. The patient's ALS-FRS score was of 26/48. Cognitive impairment was not detected, since her MMSE score was 26/30 (education: primary school).

The patient was treated with butylphthalide, vitamin B12, coenzyme Q10, and a mouse nerve growth factor. However, the patient's symptoms did not improve. Eventually, she died at the age of 58 years due to respiratory failure.

The patient recalled her mother (I-1) with similar symptoms, and possibly died of respiratory muscle weakness. Three other siblings also experienced muscle weakness and atrophy (Table 1).

**Table 1:** Clinical characteristics of patients in the 2nd generation.

Affected member	II-1	II-2	II-4	II-5
Sex	F	F	M	F
Age at onset	31	54	50	45
Age at diagnosis	-	58	51	53
Duration	4	4.5	5	9
Status	Died (35)	Died (59)	Stable	Stable
Symptoms and signs				
Initial symptom	RL	RU	RL	LU
Progression	RU, LL	LU, BL	/	RL
Pain	−	−	−	
Muscle atrophy	+	+	−	+
Muscle cramps	+	+	+	+
Muscle weakness				
Left upper extremity	×	V-/V	V/V	V/V→IV/V
Right upper extremity	×	III/V	V/V	V/V
Left lower extremity	×	V-/V	V/V	V/V
Right lower extremity	×	I/V	V-/V	V/V
Tender reflex	×	+++	+++	++→+++
Pyramidal sign	×	+	−	−
Bulbar palsy	+	−	−	−
Recognitive disorder	×	+	−	−
ALS-FRS scale	×	26	38	35
Needle EMG	×	BL	BL	BL

LU = left upper extremity, LL = left lower extremity, RU = right upper extremity, RL = right lower extremity, BL = both upper and lower extremity, x = not examined.

***II-1***: The eldest sister of the proband has already passed away for 20 years. As the proband dictated, II-1 experienced muscle weakness in her right lower limb at the age of 31 years. The symptoms deteriorated, and weakness appeared in her right arm and left leg. She was unable to walk since 31 years of age, and severe dysphagia was observed when she was 35. The patient died that year due to bulbar palsy and suspected respiratory failure.

***II-4***: A 55-year-old man with muscle weakness in his right foot since the age of 50 years He came to us half a year after onset and complained of a gradual worsening of right foot weakness and new onset of muscle cramps. No bulbar palsy, muscle atrophy, or recognition deficits were observed. EMG reflects a prolonged latent period in both the posterior tibial nerves (motor nerves). EMG suggested neurogenic lesions in both the upper and lower extremities, without clinical symptoms of muscle atrophy or weakness in either arm of the patient. The patient's condition was normal. The patient's ALS-FRS score was of 38/48.

Butylphthalide was administered as a neurotrophic treatment. At present, his symptoms are limited to both lower extremities, and he is relatively stable.

***II-5:*** She began to feel weakness and clumsiness in her left hand at the age of 45. At the age of 50, she noticed slight weakness in the right lower limb with occasional muscle cramps. We performed an intensive examination when she visited us. We noted atrophy in the left thenar eminance, although her muscle power was normal. As listed in Table 2, a prolonged latent period and reduced amplitude in the left median nerve were reported, while EMG recorded spontaneous mass activities from the left abductor pollicis brevis, suggesting a neurogenic injury to the left upper extremity. Her neurological symptoms steadily deteriorated, and she was readmitted to our hospital. Physical examination suggested decreased muscle power in the left arm and hand. The tendon reflexes were hyperactive compared to the previous time. The pyramidal signs were still negative. A follow-up EMG study reported new-onset spontaneous potentials in the right abductor pollicis brevis, left deltoid, left tibialis anterior muscle, and paraspinal muscles (Table 2). Together with the genetic outcome, the patient was eventually diagnosed and provided with active treatment. At present, patients develop obvious weakness in both hands, including disability in picking up stuffs or using chopsticks. She also complained of worsening weakness in her lower limbs, although she was still able to walk independently. The patient's cognitive function was normal throughout the course. At present, her ALS-FRS score is 35/48.

**Table 2:** Patient *II-5:* two neurophysiological examinations (2016 vs 2019).

		Latency (ms)				Amplitude (mV)				Velocity (m/s)			
		Right		Left		Right		Left		Right		Left	
Nerve Conduction		2016	2019	2016	2019	2016	2019	2016	2019	2016	2019	2016	2019
Motor													
Median	Wrist Elbow	3.4 7.9	3.7 7.6	4.1↑ 8.5↑	3.3 7.4	8.597 8.017	3.187↓ 2.926↓	4.209↓ 3.714↓	3.870↓ 4.131↓	51.1	57.6	52.2	61.4
Ulnar	Wrist Below elbow Above elbow	2.8	2.7 6.2 8.6	2.4 6.3 8.7	2.6 5.7 7.6	12.94	4.777↓ 4.753↓ 4.431↓	11.46 13.83 13.58	9.590 10.75 10.49		54.2 54.1	51.2 50.0	
Tibial	Ankle	4.7	4.3	5.1	4.7	19.47	5.537	20.29	5.823				
Peroneal	Ankle	3.1	3.3	3.2	3.0	5.628	3.116	4.233	3.298				
Sensory													
Median	Digit I Digit III	1.7 2.4	1.9 2.3	1.7 2.2	1.9 2.2	23.25 26.45	37.36 27.34	47.16 38.21	34.58 33.42	50.0 50.0	51.5 53.9	50.5 53.6	51.5 57.1
Ulnar	Wrist	1.9	1.9	1.9	1.9	34.46	22.21	23.01	20.33	53.6	59.0	49.4	54.7
Tibial	Dorsum of foot	4.4	4.4	4.5	4.3	2.528	0.703	3.371	1.071	35.9	41.6	35.2	44.1
Sural	Lower leg	2.9	3.0	3.2	2.7	22.99	19.24	18.59	26.24	46.5	49.3	46.2	50.3

		Latency (ms)				Occurrence (%)							
F-Wave Studies		Right		Left		Right		Left					
		2016	2019	2016	2019	2016	2019	2016	2019				
Median		3.4	3.7	4.5↑	3.3	95.0	95.0	0	70.0↓				
Tibial		5.1	4.3	5.3		100.0	95.0	100.0					

EMG		Spontaneous activity				Volitional MUAPs						Max volitional activity	
Muscle		Fibs		+Wave		Duration (ms)		Amplitude (uV)		Ploy (%)		Recruitment pattern	
		2016	2019	2016	2019	2016	2019	2016	2019	2016	2019	2016	2019
Sternocleidomastoid	L	−	−	−	−	10.4 (6%↑)	10.7	388	408	16.7	9.1	−	−
Deltoid	L	−	1+	−	2+	11.5 (2%↑)	12.6 (7%↑)	638 (79%↑)	721 (97%↑)	57.1	26.7	↓	↓
Abductor pollicis brevis	L	4+	1+	3+	1+	10.6 (2%↑)	12.3 (17%↑)	531 (78%↑)	620 (102%↑)	14.3	18.2	↓↓	↓
	R	−	−	−	−	10.0 (4%↓)	12.2 (16%↑)	507 (70%↑)	711 (132%↑)	0.0	0.0	↓	↓
Abductor digiti minimi (manus)	L	−	x	−	−	11.4 (4%↑)	x	443 (21%↑)	x	10.0	x	↓	x
Biceps brachii	L	−	x	−	x	11.5 (2%↓)	x	497 (47%↑)	x	36.4	x	↓	x
Tibialis anterior	L	−	−		2+	13.9 (1%↑)	13.3 (4%↓)	560 (29%↑)	691 (55%↑)	12.5	14.3	↓	↓
	R	−	−	−	1+	x	15.8 (14%↑)	x	660 (43%↑)	x	20.0	↓	↓
Vastus medialis	L	−	−	−	−	12.4 (2%↓)	13.8 (7%↑)	537 (33%↑)	600 (43%↑)	10.0	0.0	↓	↓
T10 paraspinal	L	−	−	−	−	x	x	x	x	x	x	x	x
T11 paraspinal	L	−	−	−	1+	x	x	x	x	x	x	x	x
T12 paraspinal	L	−	−	−	−	x	x	x	x	x	x	x	x

“−” = Negative, x = not examined, ↓ = decreased recruitment, ↓↓ = weak muscle contraction.

**Figure 2:**
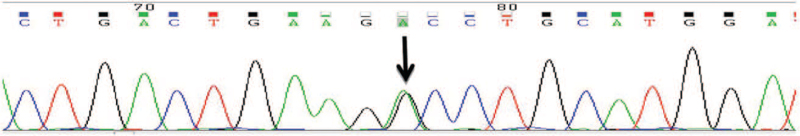
Sequencing chromatogram of the proband shows a heterozygous c.125G>A (p.Gly42Asp) missense mutation in exon 2 of *SOD1* gene.

Genetic sequencing of peripheral blood samples revealed a heterozygous c.125G>A (p.Gly42Asp) missense mutation in exon 2 of *SOD1* (Fig. 2). The 12 subjects comprised 9 carriers and 3 wild types. Three of the 9 carriers were symptomatic (II-2, II-4, and II-5), while 6 were asymptomatic (II-6, III-3, III-4, III-7, III-8, and III-9). The male-to-female ratio was 4:5 across the 2 generations of carriers. Thus, the mutation follows an autosomal dominant mode of inheritance.

## 4 Discussion

### 4.1 Phenotypic diversities exist in SOD1-mutant fALS patients

In recent decades, genetic studies have become a powerful tool for determining the etiology and pathophysiology of ALS. In 1991, Siddique established genetic correlations between a causative gene for fALS and its location on chromosome 21q22.1–22.2.^[8]^ In 1993, Rosen reported the genetic linkage between fALS and *SOD1*, a gene located on chromosome 21q that encodes superoxide dismutase for scavenging superoxide free radicals. Eleven SOD1 missense mutations were discovered in the literature within 13 isolated fALS families, including G37R, G41S, G41D, and I113T.^[5]^ Since then, more than 185 SOD1 missense mutants have been identified, with a dominant inheritance pattern in most cases.

As the mutation sites were constantly discovered, phenotypic diversity among SOD1-mutant fALS patients began to arouse attention. Familial ALS individuals with the L106 V mutation, for example, exhibited an early onset at the age of 35.5, while I113T-mutant patients showed symptoms of weakness much later (mean 58.9 years).^[9]^ Life expectancy varied according to the site of the missense mutation. The A4V-mutated cases demonstrated aggressive progression with survival shorter than 12 months; cases with H43R, G85R, or G93A mutations also showed rapid progression of disease and life expectancies shorter than 36 months from the onset. In contrast, patients with G93C or D90A mutations may have a course of more than 10 years, indicating a possible link between mutations and the degree of toxicity and pathogenicity.^[3]^ Phenotypic diversity exists among individuals sharing the same mutation pattern. For example, D90A-heterozygous patients usually have bulbar or upper-limb onset and a faster progression based on previous literature.^[10]^ However, recent studies have reported that homozygous D90A individuals with a slow progression of weakness from the legs to the arms and bulbar muscles, sensory disturbance, ataxia, or bladder disorder may occur at a later stage of disease.^[11]^

### 4.2 A literature review: G41D mutated fALS cases

In this study, we identified c.125G>A (p.Gly42Asp) as a pathogenic *SOD1* mutant in familial ALS. Therefore, we reviewed previous literature on G41D (also called G42D) mutation in fALS patients to determine its potential genotype-phenotype correlation (Table 3).

**Table 3:** A summary of G41D-mutant cases based on literature review.

Year	Number of patients	Gender	Age at onset	Duration	Single limb initiation	Bulbar palsy	Cognitive disorder
1993	1	N/A	N/A	N/A	N/A	N/A	N/A
1994	8	6M, 2F	46.8 ± 13.5	11.6 ± 1.7	√	√	X
1997	7	0M, 7F	46.0 ± 7.3	17.0 ± 6.3	N/A	N/A	N/A
2006	1	M	36	14+	√	X	X
2012	2	N/A	N/A	N/A	N/A	N/A	N/A
2015	#1	F	50	12+	√	√	√
	#2	M	46	15	√	√	√
	#3	F	50	8+	√	X	√
2017	1	M	59	1.3	√	N/A	N/A
2019	3	2M,1F	46.7 ± 38	56.7 ± 21.4	N/A	N/A	N/A

The G41D mutation was first described by Rosen in 1993.^[5]^ In 1994, Rainero reported the same mutation in an Italian family across 5 generations, including 8 patients (6 males and 2 females) and 5 carriers.^[12]^ The age at onset ranged largely from 29 to 63 years (mean age 46.8 ± 13.5), while the mean age at death was 47.8 ± 13.4. The patients exhibited a short life expectancy of less than 13 months. The phenotypes of the 8 patients were quite uniform: muscle fatigability began in a single limb and deteriorated quickly with muscle atrophy and abundant fasciculations in all 4 extremities. A bulbar deficit was observed in the late stage of the disease, and patients eventually died due to asphyxia. Later, in 1997, Cudkowicz screened 290 ALS families in the U.S. to identify SOD1 mutations.^[9]^ Seven participants from the same family were identified as G41D-carriers. All 7 individuals were female, with a mean onset age of 46. Patients had relatively longer survival (mean duration 17.0 ± 6.3 years) compared to the cohort studied by Rainero. In 2006, Stewart reported the case of a Swedish male with a G41D mutation.^[13]^ The patient showed fatigue and cramps in his left leg at the age of 36 years. No muscle weakness was observed. Hyperactive deep tendon reflexes and pyramidal signs were also observed. The patient was followed up for 14 years and was still alive at the time of the press. In 2012, Brown screened 1220 patients with ALS residing in the U.S.^[14]^ As a result, 2/92 (2.17%) of the SOD1-mutant population carried the G41D variant. However, the clinical features of the patients were not provided.

The G41D mutation was first identified in a Chinese family by Niu in 2015.^[15]^ The proband experienced muscle weakness in her left arm at 50 years of age. Muscle atrophy and cramps developed gradually. At the age of 62, she exhibited bulbar palsy symptoms, including dysarthria and dysphonia. In addition to the proband, 6 other family members underwent genetic testing. The elder brother of the proband displayed muscle weakness at age 46, developed a cognitive disorder at age 56, and died of respiratory failure by age 61. The younger sister reported weakness in her right hand at the age of 50 years and was diagnosed with 58. Four unaffected individuals from the third generation (<35 years old) were identified as G41D-carriers. In 2017, Wei screened for SOD1 mutations in 499 Chinese ALS patients and reported 1 case of a G41D variant.^[16]^ This male patient showed a strikingly rapid progression and died 16 months after onset, which was distinct from the life expectancy in most G41D-mutant cases. In 2019, Tang reported 3 cases of the G41D mutation.^[17]^ In addition, Liu et al reported 7 G41D-mutant individuals in 24 fALS cases using a screening technique.^[2]^ He claimed that the p.G41D mutation had a higher frequency in SOD1-related Chinese patients with ALS than in the Caucasian ALS cohort, indicating a possible genotypic-geographic linkage of inherited ALS.

In our study, patients from the same family showed different patterns. The proband had an onset at 54 years of age, a survival period of 5 years, early manifestation of right-hand muscle weakness, and mild symptoms within 4.5 years since the onset. Electrophysiological abnormalities at the early stage were confined to the right upper limb, which could account for the misdiagnosis of the disease and partly lead to later rapid progression with extensive neurogenic damage and poor prognosis. The proband's younger sister, II-5, had an earlier age of onset (45 years) and a longer course of disease (9 years, still alive) without problems in walking or breathing. The proband's brother, II-4, developed lower extremity weakness as his initial symptom. He was diagnosed in the 2nd year after onset and received timely treatments; thus, his symptoms were still limited to the lower limbs. The proband's younger sister II-6 is now 48 years old. As a mutation carrier at a vulnerable age, the patient showed no symptoms. Unlike the pedigree reported by Niu in 2015, none of our patients exhibited cognitive disorders, which is further evidence for phenotypic diversity.

### 4.3 Genetic sequencing is a powerful tool for early diagnosis of ALS

Our proband experienced several misdiagnoses throughout the course of ALS owing to her atypical EMG manifestations. It was already too late for genetic sequencing to provide a definitive diagnosis. The same scenario occurred to the proband's siblings II-4 and II-5 when they came to us. As shown in Table 2, we were unable to provide a diagnosis of ALS to II-5 (II-4 as well) in 2016 based on her NCV and EMG findings. However, precisely because of genetic sequencing, the patients were diagnosed and provided timely treatment.

Genetic sequencing is a common tool for the diagnosis of inherited diseases. Through the study of our pedigree, we sincerely emphasize the significance of genetic testing of suspected fALS families, even members that have no symptoms yet. We are still following up, hoping to support the family as early as possible. In addition, we are currently trying to understand the pathogenesis of ALS. For example, protein-RNA and protein-protein interactions are key to the ALS process. Therefore, proteomic studies may help unevil the mysteries of phenotypic heterogeneity, which is a part of our future research.

## 5 Conclusion

Here, we report a case of Chinese fALS with a heterozygous missense mutation, c.125G>A (p.Gly42Asp). A literature review has suggested phenotypic heterogeneity among individuals. Genetic sequencing provides evidence for diagnosis; thus, it is strongly recommended that it be performed before or at early onset of the disease. Our next step is to conduct a functional study of the G41D mutation to gain a deeper understanding of fALS pathogenesis and provide targets for treatment.

## Acknowledgments

We sincerely thank the participants of this study.

## Author contributions

**Conceptualization:** Xinyi Zhao, Xinhong Feng.

**Data curation:** Xinyi Zhao, Xiuli Li, Jingyu Mou.

**Formal analysis:** Xinhong Feng.

**Methodology:** Hongjing Liu, Jing Chen, Xinhong Feng

**Supervision:** Jian Wu, Xinhong Feng.

**Funding acquisition:** Jian Wu.

**Writing – Original draft:** Xinyi Zhao.
